# Host-like RNA Elements Regulate Virus Translation

**DOI:** 10.3390/v16030468

**Published:** 2024-03-20

**Authors:** Debjit Khan, Paul L. Fox

**Affiliations:** Department of Cardiovascular and Metabolic Sciences, Lerner Research Institute, Cleveland Clinic, Cleveland, OH 44195, USA

**Keywords:** internal ribosome entry sites, RNA element, SARS-CoV-2, translation control, untranslated region, upstream open reading frames, virus, virus-host interaction

## Abstract

Viruses are obligate, intracellular parasites that co-opt host cell machineries for propagation. Critical among these machineries are those that translate RNA into protein and their mechanisms of control. Most regulatory mechanisms effectuate their activity by targeting sequence or structural features at the RNA termini, i.e., at the 5′ or 3′ ends, including the untranslated regions (UTRs). Translation of most eukaryotic mRNAs is initiated by 5′ cap-dependent scanning. In contrast, many viruses initiate translation at internal RNA regions at internal ribosome entry sites (IRESs). Eukaryotic mRNAs often contain upstream open reading frames (uORFs) that permit condition-dependent control of downstream major ORFs. To offset genome compression and increase coding capacity, some viruses take advantage of out-of-frame overlapping uORFs (oORFs). Lacking the essential machinery of protein synthesis, for example, ribosomes and other translation factors, all viruses utilize the host apparatus to generate virus protein. In addition, some viruses exhibit RNA elements that bind host regulatory factors that are not essential components of the translation machinery. SARS-CoV-2 is a paradigm example of a virus taking advantage of multiple features of eukaryotic host translation control: the virus mimics the established human GAIT regulatory element and co-opts four host aminoacyl tRNA synthetases to form a stimulatory binding complex. Utilizing discontinuous transcription, the elements are present and identical in all SARS-CoV-2 subgenomic RNAs (and the genomic RNA). Thus, the virus exhibits a post-transcriptional regulon that improves upon analogous eukaryotic regulons, in which a family of functionally related mRNA targets contain elements that are structurally similar but lacking sequence identity. This “thrifty” virus strategy can be exploited against the virus since targeting the element can suppress the expression of all subgenomic RNAs as well as the genomic RNA. Other 3′ end viral elements include 3′-cap-independent translation elements (3′-CITEs) and 3′-tRNA-like structures. Elucidation of virus translation control elements, their binding proteins, and their mechanisms can lead to novel therapeutic approaches to reduce virus replication and pathogenicity.

## 1. Introduction

**Figure 1:**
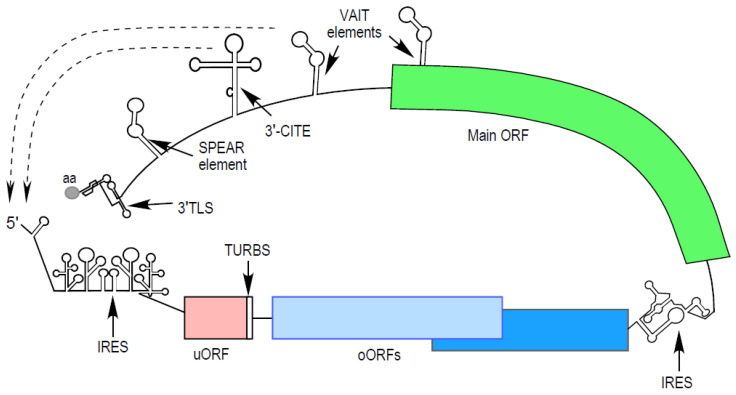
Translation in viruses is regulated by themes shared with hosts. Various RNA elements shown here are IRES (Internal Ribosome Entry Site), uORF (upstream Open Reading Frame), oORF (overlapping Open Reading Frame), TURBS (Termination Upstream Ribosome Binding Site), VAIT (Virus Activated Inhibitor of Translation element), 3′-CITE (3′-Cap Independent Translation Enhancer), SPEAR (Sarbecoviral Pan-End Activating RNA element), and 3′TLS (3′ tRNA-Like Structure) that can be charged with amino acid (aa). IRESs and 3′-CITEs are diverse, as discussed in the text. 3′-CITEs and VAIT activate and inhibit virus translation in a 5′-end dependent manner, as shown with dashed lines.

The flow of genetic information in biological systems is tightly regulated at all levels, that is, at replication, transcription, and translation. Post-transcriptional gene regulation allows rapid biological responses when challenged by cellular signals and environmental stimuli. A principal component of post-transcriptional gene regulation is translational control [1,2,3]. mRNA translation occurs in four stages: initiation, elongation, termination, and ribosome recycling. Translation initiation is generally the rate-limiting step in protein synthesis and is exquisitely regulated by a variety of mechanisms [1,2,4]. In eukaryotes, translation of most mRNAs is initiated via cap-dependent scanning [5,6]. Viruses are obligate, intracellular parasites that co-opt host cell machineries for propagation. Eukaryotic viruses subvert host translation machinery by employing diverse and elaborate RNA-centered strategies, often mimicking host features [7]. Utilizing such features that draw upon shared resources, viral RNAs can compete with, and even outpace, host mRNAs for selfish benefits. In this review, we present the RNA elements (Figure 1) and their protein partners that are key to shared virus and host translational control mechanisms underlying the pathogenesis of viral diseases and are targetable with a synthesis of understanding of the molecular interactions.

## 2. Virus 5′ End Elements

An archetype of eukaryotic translation regulation was initially described about 50 years ago in mouse liver extracts, where ferritin mRNA was induced by iron [8]. In the absence of iron, iron responsive elements (IRE) containing 25- to 30-nt stem-loop secondary structures in the ferritin and eALAS mRNA 5′-untranslated regions (UTRs) were shown to recruit iron regulatory protein 1 (IRP1) and block scanning of the 43S pre-initiation complex, and thus translation [9,10,11]. In the presence of iron, the IRP1 does not bind the IRE, thus permitting scanning and translation and representing a paradigm of 5′-end-directed translation regulation.

### 2.1. Internal Ribosome Entry Sites

Parallel to the landmark studies on the eukaryote IRE, pioneering studies were reported on viral translation regulation through internal ribosome entry sites (IRESs) (Figure 1). Increasing secondary structure complexity and content in 5′ UTRs of eukaryotic mRNAs, as well as viral RNAs, generally decreases translation efficiency [12]. In contrast, IRESs are RNA elements, defined by structure, sequence, or both, that recruit ribosomes at internal regions of mRNAs to initiate translation. IRESs were first discovered in picornaviruses such as poliovirus (PV) and encephalomyocarditis virus (EMCV) [13,14]. Soon afterwards, human immunodeficiency virus (HIV), hepatitis C virus (HCV), and foot and mouth disease virus (FMDV) IRESs were reported [15,16,17], and the inventory of viruses utilizing IRESs continues to expand [18]. Although the viral IRESs contain a diversity of sequences, many have similar secondary structures and initiate translation through similar mechanisms. Importantly, viruses and host cells often share translation control mechanisms, and cellular IRESs were soon reported in mRNAs encoding proteins required in physiological and pathological stress responses, e.g., apoptosis, hypoxia, and starvation [19,20,21,22,23,24]. Generally, cellular IRESs are not well conserved in either sequence or structure and are difficult to predict from sequence ab initio, and require rigorous experimental verification [25,26,27,28,29]. Notwithstanding, high-throughput methods such as mRNA display have revealed the genome-wide presence of cap-independent translation-enhancing elements [30], and nearly 10% of mammalian mRNAs contain RNA elements that potentially function as IRESs [31]. The scope and challenges in cellular IRES annotation and validation have been described in detail [32]. Mis-annotation of 5′UTRs, cryptic promoter activity, presence of 5′ introns and alternate splice sites, confusion of cap-independence with 5′end-independence, and unnatural experimental platforms have led to contested interpretation of cellular IRESs [33,34], and an essential workflow for characterizing cellular IRESs has been postulated [35]. Many viral IRESs function completely independent of the 5′-terminal cap structure, while cap-dependence of some viral IRESs and many cellular IRESs is more nuanced and context-dependent [32,36,37,38,39,40,41]. In addition to common translation initiation factors (eIFs), IRESs often recruit IRES-specific RNA-binding proteins, i.e., IRES trans-acting factors (ITAFs), for translation initiation (reviewed in [20,42,43,44]).

Many viruses, including picornaviruses, deploy a variety of strategies directing global shutdown of host cap-dependent translation while the IRESs promote translation of viral RNAs, thereby conferring a competitive advantage to the virus [7,45,46]. For example, PV infection cleaves eIF4G, preventing end-to-end circularization and translation of host mRNAs, while the IRES utilizes the cleaved carboxy-terminal fragment of eIF4G that binds eIF3 for translation initiation. In PV and EMCV infections, eIF4E is sequestered by hypophosphorylated 4E-BP, reducing cellular cap-dependent translation, without affecting IRES-dependent viral translation [47]. In both cases, viral infection essentially frees up the cellular translation machinery for self-serving protein synthesis.

### 2.2. Class 1 and 2 IRESs

Viral IRESs can be grouped into four classes by structure and the requirement of eIFs and ITAFs. In general, Class 1 and 2 IRESs do not bind the 40S subunit directly and require all canonical translation initiation factors, except the cap-binding protein eIF4E [48,49]. They generally present as complex secondary structures comprised of multiple domains, each containing stem-loops, bulges, and junctions. These structures are recognized by ITAFs, facilitating eIF binding and 43S pre-initiation complex recruitment. Long-range RNA–RNA interactions are also involved in the function of these IRESs [50,51]. Despite their similarity in length and multi-domain RNA structure, Class 1 and Class 2 IRESs are not identical in their secondary structures, ITAF requirements, or mechanism of initiation (reviewed in [39,48]); however, ribosomal subunit scanning after internal recruitment of the pre-initiation complex may not be considered a distinguishing factor between the two classes. For example, initiation on Class 1 IRESs can occur at the IRES-adjacent upstream codon as well as downstream at the polyprotein initiation codon [52,53,54]. Similarly, initiation on Class 2 IRESs can occur at the IRES-adjacent codon but also occurs downstream in all FMDV isolates [55,56], and in Theiler’s murine encephalomyelitis virus (TMEV), downstream initiation yields the L* protein by translation of an overlapping ORF [57,58]. Class 1 and 2 IRESs are partially or completely refractory to translation inhibition by eIF2α phosphorylation [59,60], suggesting they do not require canonical Met-tRNAi^Met^ delivery by the ternary complex. In general, eIF2-less initiation can be facilitated by eIF2A [61], eIF2D (Ligatin) [62], eIF5B [63], or by the concerted action of MCT-1 and DENR [64]. Multiple ITAFs have been identified for the Class 1 and 2 IRESs; the specific ITAF required is variable [42,43,46]. The Class 2 EMCV IRES binds the ITAF polypyrimidine tract-binding protein (PTB), thus altering RNA conformation [65,66]. The Class 1 PV IRES binds both PTB and poly(rC)-binding protein 2 (PCBP2) as ITAFs [65,67]. Domain V in PV IRES binds cellular glycyl-tRNA synthetase (GARS), promoting association of the IRES in the mRNA binding site of the ribosome [68]. Cellular Unr protein is required for human rhinovirus (HRV) IRES activity and acts synergistically with PTB, while such synergy is absent for PV IRES, highlighting differential ITAF requirements within the same IRES class [69].

### 2.3. Class 3 IRESs

Class 3 IRESs, exemplified by the hepatitis C virus (HCV) of the family Flaviviridae, are present at the 5′ end of viral RNAs and contain multiple stem-loop structures organized around helical junctions and a pseudoknot with additional requirements of ITAFs, e.g., PCBP2 and La [70,71,72]. Class 3 IRESs require a small set of canonical eIFs and a notable ability for eIF2-less initiation [63,73], but can operate in either eIF2-dependent or eIF2-independent modes depending on availability [62,64,74,75,76]. Incoming 40S subunits take a “land and initiate” approach; the stem-loop structures directly capture the ribosome at the AUG start codon, thereby bypassing scanning [77,78,79]. The 40S ribosomal subunit and eIF3 bind and are stabilized by domains II and III of the IRES RNA [73,80]. The interaction of eIF3 and 40S with the IRES is structurally distinct from the cap-dependent scanning mode of translation initiation [78,79,81,82,83,84]. HCV IRES subdomain IIIb as well as a stem-loop in the specialized translation initiation element in the cellular *c-JUN* transcript 5′UTR share structural similarity and bind eIF3d [85]. Nonetheless, recruitment of the 60S subunit leads to 80S ribosome formation and subsequent elongation [77,86,87]. An extensive catalog of HCV IRES-40S interactions has been described [88]. This study also demonstrates how eIF5B reorients initiator tRNA prior to ribosome subunit joining, promoting eIF2-independent initiation.

### 2.4. Class 4 IRESs

Class 4 IRESs have been identified to date only in the Dicistroviridae family of picornaviruses, e.g., cricket paralysis virus (CrPV). The single-stranded viral RNA genome contains two open reading frames (ORFs) separated by an intergenic region (IGR) that harbors an IRES [89,90] (Figure 1). Class 4 IRESs are considered “factorless”, that is, without the requirement of eIFs and ITAFs, yet display strong translation initiation potencies in multiple cell types [91,92,93,94]. These IRESs are folded into a compact, ordered three-dimensional structure consisting of multiple pseudoknots and stem-loops that directly interact with the 40S ribosomal subunit and permit assembly of the 80S ribosome [95,96,97]. Moreover, the IRES structure can directly recruit an 80S ribosome in vitro [98]. Remarkably, the IRES initiates translation from a non-AUG start codon, bypassing the requirement for the eIF2/Met-tRNAi/GTP ternary complex [97,99,100]. An RNA pseudoknot in the Class 4 IRES mimics a cellular tRNA that occupies the ribosome P-site and directs a tRNA-less elongation step, i.e., pseudotranslocation, poising translation to start from the second elongation step [91,101,102,103,104,105]. eIF2α phosphorylation, a hallmark of viral infections and other stresses, can increase translation efficiency of these IRESs, thereby flipping this antiviral response mechanism to the benefit of the virus [106].

### 2.5. Hybrid IRESs and Other IRESs

In many viruses, IRESs operate outside the bounds of easy classification into the schemes outlined above. Uniquely, the hepatitis A virus IRES depends on eIF4E, requiring its association with eIF4G to unwind RNA [107,108]. A “Class 5” IRES was identified in *Kobuvirus*, *Salivirus*, and *Paraturdivirus* genera of Picornaviridae [109]. Various domains in this IRES class, as typified in Aichivirus, are shared with both Class 1 and Class 2 IRESs, with a unique requirement of cellular DHX29 protein as an ITAF [109]. IRESs that are not classified within the four common types are present in pegiviruses, pestiviruses, and hepaciviruses [110]. Retroviral IRES function, including HIV-1 IRES, is debated as viral transcripts are capped [38,111,112]. In addition, a specialized translation mechanism of HIV-1 mRNAs sustained during eIF4E repression can be directed by a hypermethylated cap instead of being cap-independent, requiring interaction of the primer binding site in viral 5′UTR with cellular nuclear RNA helicase A (RHA)/DHX9 [113]. The IRES in the iflavirus *Ectropis obliqua* picorna-like virus (EoPV) is similar in several functional and structural aspects to Class 2 IRESs in cardioviruses and aphthoviruses; however, unlike Class 2 IRESs, it lacks a GNRA motif, has a functionally redundant RAAA motif, and utilizes a non-homologous initiation codon [114]. The dicistrovirus CrPV 5′UTR-IRES assembles a functional initiation complex utilizing an upstream start codon within the IRES [115]. In another dicistrovirus, the Halastavi árva virus (HalV), the 5′UTR IRES utilizes an unusual retrograde scanning of the 43S complex for initiation [36].

In summary, IRES structures are diverse and employ equally diverse mechanisms with limited common principles that serve as a framework for features specific to a single IRES or IRES class. Focused reviews contain further information on viral IRESs [39,46,48,49,116]. Beyond IRESs, viral and cellular transcripts can share specialized themes in cap-dependent translation initiation as well, e.g., cis-acting elements in the 5′UTR of transcripts from *Mononegavirales* family members such as vesicular stomatitis virus (VSV), measles virus, and rabies virus, as well as cellular transcripts such as *DDR2*, interact uniquely with RPL40 (ribosomal protein 40, large subunit) for cap-dependent translation initiation, suggesting these viruses might have usurped an endogenous translation pathway [117]. It remains to be seen if other alternative pathways of cap-dependent cellular translation initiation, e.g., eIF3d-dependent [118] and eIF4EHP-dependent [119], are utilized by multiple viruses.

## 3. Upstream Open Reading Frames

### 3.1. Translation Control by Upstream Open Reading Frames

A series of landmark studies revealed translation repression of *GCN4* mRNA in brewer’s yeast (*Saccharomyces cerevisiae*) through initiation events at small ORFs in its 5′UTR [120,121,122,123]. Upstream open reading frames (uORFs) are *cis*-regulatory RNA elements prevalent in eukaryotic mRNAs that regulate translation initiation of downstream coding sequences (CDSs) (Figure 1). The pre-initiation complex (PIC) scans the 5′ UTR for a start codon until it recognizes an upstream AUG (uAUG), thereby permitting assembly of 80S ribosomes at the uORF. uORFs serve as “roadblocks”, translation elongation following uORF recognition prevents PIC scanning to the downstream AUG of the main ORF. The Kozak sequence context of an uAUG, as in a canonical CDS, is a critical determinant of translation initiation of an uORF (reviewed in [124]). A weak context at the uAUG leads to “leaky scanning” by 43S PIC and initiation at the main ORF, while a strong context results in translation elongation at the uORF, necessitating re-initiation events by 40S subunits at the downstream AUG of the main ORF. Non-canonical initiation codons, e.g., CUG, GUG, and UUG, can also direct upstream initiation at uORFs [125,126]. uORFs attenuate main ORF translation by multiple mechanisms: by the “first dibs” scheme of translation initiation, by ribosome dissociation at the uORF stop codon, by stalling-mediated mRNA decay, and by encoding regulatory peptides [4,127,128,129]. uORF-mediated repression is widespread across the mammalian transcriptome [130,131,132] and is associated with diseases and stress responses (reviewed in [133,134,135,136]). For example, the integrated stress response depletes pre-initiation complexes to specifically de-repress translation from uORF-containing mRNAs [137,138,139]. A PIC reinitiates at a main ORF initiation site only when an uORF does not overlap with the main ORF. In contrast, an upstream AUG preceding the main ORF start codon can be in an overlapping ORF, with 2 further subclasses: out-of-frame overlapping uORFs (oORFs), the stop codons of which are downstream of main ORF AUGs and in different reading frames, and N-terminal extensions, which are basically overlapping uORFs in-frame with the main ORF [140,141,142,143,144] (Figure 1). The downstream CDS can be engaged by the re-initiating PIC only when the translated uORFs do not overlap with CDSs [145], or in special cases through a TURBS (termination upstream ribosome binding site) RNA element at the end of the preceding ORF (reviewed in [145,146], see below) (Figure 1). In addition to leaky scanning, bi-directional scanning of the PIC between closely spaced start codons, as in oORFs, can influence start site selection [147,148].

### 3.2. Virus Utilization of uORFs and uAUGs

Viruses take advantage of oORFs to offset genome compression and increase coding capacity [149]. Likewise, utilization of uORFs and uAUGs is commonplace in DNA viruses as well as in positive- and negative-sense RNA viruses [150,151]. Examples representing multiple mechanisms and viruses are summarized here.

Hepatitis B virus (HBV) polymerase ORF is preceded by an overlapping core ORF and by a more upstream AUG in the viral pre-genomic RNA [152]. Translation from the uAUG represses translation of the core ORF while allowing reinitiation of translation at the polymerase ORF [153,154]. Translation of the Human Cytomegalovirus (HCMV) gpUL4 ORF is regulated by an uORF [155]. Kaposi’s sarcoma-associated herpesvirus (KSHV) ORF35 and ORF36 are translated from a polycistronic transcript regulated by a pair of uORFs. The second uORF overlaps with ORF35 and allows translation of ORF36 by a reinitiation mechanism, which is essential for viral propagation [156,157]. An uORF in the ebolavirus (EBOV) L gene suppresses translation of the L ORF under normal conditions and increases it under stress to maintain optimal polymerase activity, and uORF mutations attenuate EBOV growth [158]. Human immunodeficiency virus type 1 (HIV1) *tat* ORF is followed by *rev* and *nef* ORFs, and translation of upstream *tat* strongly represses Rev production [159]. A minimal uORF, containing only a start and a stop codon within the HIV1 *vpu* leader, permits efficient translation initiation at the downstream *env* start codon in viral vpu-env bicistronic mRNAs [160]. In arteriviruses, an uORF is present in the 5′-leader of the genomic RNA (gRNA), as well as in all sub-genomic RNAs (sgRNAs). In related coronaviruses, the uORF maps downstream of the genomic leader and is present only in the gRNA [161]. Bovine coronaviruses are an exception in which the uORF is utilized in all sgRNAs during persistent infection, causing translation attenuation [162]. Simian immunodeficiency virus (SIV) Rev and Env proteins are regulated by up to five uAUGs in multiple splice variants [163,164]. uORF translation enhances ribosomal shunting in prototype foamy virus genomic RNA [165] and in rice tungro virus [166], in a way that enables the mRNA or viral RNA to escape translation inhibition.

Termination-reinitiation events in uORF/main ORF pairs are regulated by TURBS elements extensively characterized in viruses of the Caliciviridae family and in influenza B virus from the Orthomyxoviridae family [167,168,169]. In these cases, the uORF stop codon and downstream ORF start codon are in close proximity, out-of-frame, or overlap in consecutive nucleotides. The viral TURBS regulates reinitiation through base-pairing with 18S rRNA and tethering of the post-termination 40S subunit (reviewed in [145,146]). It has been suggested that uORFs in the 5′UTR regulate translation of cellular *SLAMF1* mRNA utilizing a TURBS-like element, highlighting shared strategies in eukaryotic translational control [170]. Additionally, functional polypeptides encoded by viral uORFs can regulate virulence and tropism. For example, a conserved uORF overlaps the viral polyprotein ORF in enteroviruses, including echovirus 7 and poliovirus 1, encoding a transmembrane protein that facilitates virus growth in gut epithelial cells [53].

## 4. 3′ End Elements

### 4.1. GAIT and VAIT Elements

The Gamma-interferon-Activated Inhibitor of Translation (GAIT) RNA element was first identified in the 3′UTR of human ceruloplasmin mRNA as an inducible, translation repressive element [171]. Subsequent studies observed similar structurally conserved GAIT elements in *VEGFA*, *DAPK*, and *ZIPK* mRNAs. To date, translation-repressive GAIT elements have been reported in multiple inflammation-related human mRNAs [171,172,173,174]. The GAIT RNA element is characterized by a split stem-loop secondary structure, with generally conserved A and U residues (absent in the *ZIPK* GAIT element [172]) in an asymmetric bulge separating the stems. The GAIT element recruits a gamma-interferon-inducible, heterotetrameric complex in which the direct RNA-interacting constituent is the bifunctional glutamyl-prolyl tRNA synthetase EPRS1 [175,176]. EPRS1 contains two catalytic domains joined by a linker containing three repeats of an atypical RNA-binding domain termed the WHEP domain, also found in human tryptophanyl, histidyl, glycyl, and methionyl tRNA synthetases. Three other constituents of the human GAIT complex are ribosomal protein L13a, heterogenous ribonucleoprotein Q (or NSAP1), and GAPDH. Eukaryotic translation occurs in a closed loop where the 5′ and 3′ ends of translated RNAs are generally bridged by eIF4G and PABP interactions [177,178,179]. L13a is central to the repressive function of the GAIT complex, as it blocks eIF4G from recruiting the pre-initiation complex [180].

The GAIT system is a classical archetype of a post-transcriptional regulon in which families of functionally related mRNAs are co-regulated by specific RNA-binding proteins that target similar sequences or structural elements [181,182]. Mimicking host RNA structures, GAIT-like RNA elements have been described in the RNA viruses RSV (respiratory syncytial virus) and the pig alphacoronavirus TGEV (transmissible gastroenteritis virus); both repress viral RNA translation in response to viral cues [183,184]. The cellular GAIT element, the RSV GAIT-like element binds L13a and has been termed a VAIT or Virus-Activated Inhibitor of Translation element [184] (Figure 1). Two coding-region VAITs have been reported in SARS-CoV-2, one in ORF1a of the genomic RNA and another in the coding region of Spike subgenomic (sgRNA) [185]. Unlike the VAITs, the inhibitory TGEV GAIT-like element does not bind L13a but binds two aminoacyl-tRNA synthetases (aaRSs), EPRS1 and RARS1, showcasing L13a-independent translation repression [183], and suggesting similar RNA structural motifs can recruit distinct but functionally similar machineries.

### 4.2. Sarbecoviral Pan-End Activating RNA Element

**Figure 2:**
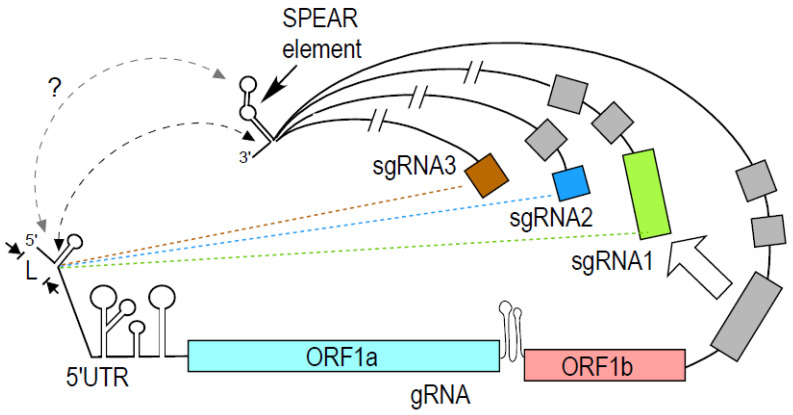
A unique regulon in viruses that employ discontinuous transcription can regulate genomic and sub-genomic RNAs. As an example, a simplified schematic of the SPEAR regulon of SARS-CoV-2 is shown. L: 5′-leader (within solid black arrows) that resides in the 5′UTR, gRNA: genomic RNA, sgRNA: subgenomic RNA. Frameshift element between ORF1a and ORF1b is shown in grey. Green, brown and blue dashed lines represent sgRNA-generating events between identical transcription regulatory sequences contained in 5′-leader and in nucleotides 5′ to ORFs encoded in sgRNA1 (green), sgRNA2 (blue), sgRNA3 (brown) and so on. The genomic terminus, common to gRNA and sgRNAs, reportedly pairs with the 5′-leader (darker gray dashed line). 5′-3′ communication through the SPEAR element is yet unknown (lighter gray dashed line).

We reported a novel 39-nucleotide GAIT-like RNA element in the 3′-end of SARS-CoV-2 genomic and sgRNAs [186] (Figure 1). Discontinuous transcription of the viral gRNA generates an ensemble of nested 3′-co-terminal sgRNAs that contain 5′-leader and 3′-end sequences identical to each other and to the genomic RNA (gRNA) (Figure 2). Thus, the element defines a unique post-transcriptional regulon distinct from non-viral systems, in which the elements are structurally related but not identical in sequence [187]. The RNA sequence is conserved in viruses of the subgenus Sarbecovirus of the genus Betacoronavirus, including SARS-CoV-1, suggesting an invariant function. Host-derived insulin and IFN-γ, agents associated with COVID-19 severity and outcome, singly or additively with SARS-CoV-2 spike subunit 1 that contains the host ACE2-receptor binding domain, increase sgRNA expression and translation only when the cis-element is intact [186]. Therefore, the element was termed the Sarbecoviral Pan-End Activating RNA (SPEAR) element. Strikingly, EPRS1 binds SPEAR as part of an unconventional, inducible Tetra-Aminoacyl-tRNA synthetase Sarbecoviral RNA-Interacting (TASRI) complex, containing three other aaRSs: arginyl-, lysyl- and methionyl-tRNA synthetases (RARS1, KARS1, and MARS1, respectively). Of note, SPEAR, such as the repressive TGEV GAIT-like element, does not bind L13a, L13a-interacting RSV, or SARS-CoV-2 VAITs [184,186]. Binding of RARS1 and EPRS1 to the repressive TGEV element [183] suggests the translation-enhancing function of SPEAR might depend on the other binding partners, i.e., KARS1 or MARS1, or both. Potential internal initiation at ORF10, a 3′-end co-terminal feature newly acquired in SARS-CoV-2, appears to play a critical role in SPEAR-mediated induction of viral sgRNA expression [186]. The SPEAR element and TASRI complex together form a host system hijacked by SARS-CoV-2 to direct an outcome, i.e., translation enhancement, opposite to the original host function of translation inhibition.

Surprisingly, the SPEAR element at the 3′-end of gRNA supports ribosomal frameshifting in reporter assays, but only in the presence of the genomic 5′UTR [186], indicative of a 5′-end-dependent role of the SPEAR element. Circularization of eukaryotic mRNAs by interactions of proteins bound to both termini have been proposed to augment translation efficiency, possibly by facilitating ribosome transfer to the initiation site following a round of elongation [177]. In an early example of mRNA circularization influencing translation control, rather than translation efficiency, translational silencing by the 3′UTR GAIT element was shown to require the elements of mRNA circularization, including eIF4G [188,189]. In an alternative control mechanism that takes advantage of the closed loop, the 3′-Cap-Independent Translation Elements (CITEs, described in detail in Section 4.3), structurally diverse elements in the 3′UTRs of many plant viral genomes facilitate translation by recruiting initiation factors, ribosomes, and ITAFs to the 3′UTR, which exhibit trans-terminal effects on initiation at the 5′-end [116,190,191,192,193,194,195]. Indeed, SARS-CoV-2 initiates a global shutdown of translation, possibly necessitating a CITE-like function of SPEAR [196]. A proposed closed-loop mechanism placing the SPEAR element in proximity of the start codon across all viral sgRNAs awaits verification [197]. The 5′UTR of SARS-CoV-2 gRNA exhibits cap-independent translation activity; however, the smaller 5′-leaders of sgRNAs are eIF4E-dependent [198]. Since the SPEAR element is present in all SARS-CoV-2 RNAs and might facilitate 5′-3′ end communication, the difference in translation-initiation modes between 5′UTR of gRNA and 5′-leaders of sgRNAs can further indicate diverse roles of SPEAR in the 5′-3′ communications of gRNA compared to sgRNAs (Figure 2).

A critical feature of the discontinuous transcription program utilized by SARS-CoV-2 is the generation of an ensemble of nested 3′-co-terminal sgRNAs containing 5′ leader and 3′ end sequences identical to each other and to the genomic sequence [186]. Thus, the system forms a “post-transcriptional regulon” analogous to those found in eukaryotes, in which families of functionally related mRNAs are co-regulated by specific RNA-binding proteins that target similar sequences or structural elements [181]. However, the viral system is distinct in that the RNAs regulated are contained within the same genetic unit, versus scattered among chromosomes in eukaryotic systems. Also, the element is identical in the RNAs of the viral system, compared to the similarity of sequence or structure in eukaryotic systems. The “thrifty” virus mechanism that compacts the genome, i.e., reusing the genomic termini in every sgRNA, can be turned against the virus as a therapeutic strategy. Thus, interfering with the SPEAR-TASRI complex interaction is an attractive antiviral strategy to target the entire SPEAR regulon. Importantly, interception of the SPEAR element/TASRI complex interaction with peptide-conjugated phosphorodiamidate morpholine oligonucleotides (PPMOs) antisense to the SPEAR element markedly reduces SARS-CoV-2 replication in Caco-2-hACE2 cells [186].

### 4.3. 3′-Cap-Independent Translation Elements

The trans-terminal-acting 3′-CITEs are structurally diverse elements in seven identified classes [39] (Figure 1). The 5′ region of the viral RNA is essential for the function of certain 3′-CITEs through 5′-3′ communication, even if the cap is not [199,200,201] (Figure 1). These elements can compete with cellular mRNAs for the eIFs; e.g., barley yellow dwarf virus translation element (BTE) binds eIF4G [202,203,204] and panicum mosaic virus-like translation enhancers (PTEs) bind eIF4E while base-paired with the 5′UTR [205,206]. Crystal structures have shown that a guanosine is extruded from a G-rich stretch in the PTE of the pea-enation mosaic virus (PEMV2), providing a structural basis for eIF4E recognition [207]. Similar studies in the saguaro cactus virus (SCV) revealed a flipped-out G residue ready to dock into the 5′ cap-binding pocket of eIF4E, highlighting 5′ cap mimicry by a 3′-CITE [208]. 3′-CITEs in T-shaped structures (TSSs) in TCV and cardamine chlorotic fleck virus RNAs can recycle post-termination ribosomes or ribosomal subunits directly to the 5′-end [209,210]. The 55-nt Cucurbit aphid-borne yellows virus-Xinjiang-like translation element (CXTE) functions in eIF4E-depleted lysate and is enhanced by the viral 5′UTR; however, detailed molecular mechanisms remain unknown [211]. Interfamilial recombination of 3′-CITEs between viruses has been observed, and they can act in combination with other elements, e.g., the melon necrotic spot virus has a CXTE and an I-shaped structure (ISS) [211], and the PTE and an upstream TSS in PEMV2 [212]. Furthermore, 3′-CITEs are conformationally dynamic [213], e.g., the TCV TSS is disassembled through viral RNA-dependent RNA polymerase binding, a requirement for replication [214]. Structural rearrangement is also proposed for the ORF10-dependent function of the SPEAR element in SARS-CoV-2 sgRNA 3′-end, comparable to 3′-CITEs [186,197]. The TSSs in TCV and PEMV2 are predicted to form internal tRNA-like structures (TLS), ubiquitous host molecules linking the amino acid and RNA worlds [192].

### 4.4. 3′-tRNA-Like Structures

The plant RNA virus Tobamovirus and Tymovirus genomes contain 3′-terminal TLSs that control their replication [215,216,217] (Figure 1). Like the aaRS-interacting GAIT and TGEV elements, these 3′-TLSs recruit specific aaRSs and are aminoacylated with histidine (e.g., tobacco mosaic virus, TMV), tyrosine (e.g., brome mosaic virus, BMV), or valine (e.g., turnip yellow mosaic virus, TYMV) to stabilize viral RNAs, enhance 5′-cap-dependent translation by communicating with 5′UTR elements in the closed-loop model of translation initiation, and possibly recruit eIFs and ribosomes [215,218,219]. The TLS in TYMV interacts with an upstream pseudoknot domain, possibly constituting a longer, modular structural element that increases the stability of this RNA substrate for valylation [220]. It remains contentious whether the TLS delivers valine as the first amino acid for viral protein synthesis [221,222]. The BMV TLS binds tyrosyl-tRNA synthetase in a conformation that markedly differs from a tRNA [223], further illustrating the dynamics of viral RNA structures. The TLS of the *Hordeivirus* genus in Virgaviridae, although structurally distinct from BMV TLS, can be tyrsylated, and, more importantly, a chimeric *hordeivirus*-BMV TLS can be similarly aminoacylated, illustrating modularity in TLSs [224]. Likewise, tRNA mimicry is seen in 5′-localized structures in viral RNAs, e.g., poliovirus IRES domain V resembles glycine tRNA and binds glycyl-tRNA synthetase for translation initiation [68].

## 5. Conclusions

Viruses employ a diverse array of host-directed translational strategies for efficient expression of viral proteins. Lacking the essential machinery of protein synthesis, for example, ribosomes and other translation factors, all viruses take advantage of the host apparatus to generate virus protein. Layered above the absolute requirement for the translation machinery, viruses have adopted certain features of host translation control to the benefit of the virus. In some cases, the mechanism itself is imitated; for example, virus utilization of IRESs and uORFs is used to increase coding capacity while reducing genome size. Some viruses have co-opted eukaryotic sequence or structural elements generally employed for condition-dependent regulation, often translation repression. Viruses can incorporate similar elements to recruit essential constituents of the eukaryote translation apparatus, generally to enhance translation initiation. Other virus elements recruit host regulatory factors that are not components of the translation machinery. The utilization of multiple aaRSs as trans-acting factors interacting with a variety of virus elements forms an unexpected molecular leitmotif. SARS-CoV-2 is a paradigm example of a virus conjoining several distinct features of eukaryotic host translation control: the virus mimics an established regulatory element and co-opts four aaRSs to form a stimulatory binding complex. Utilizing discontinuous transcription, identical elements are present in all SARS-CoV-2 sgRNAs (and the gRNA). Thus, the virus post-transcriptional regulon improves upon comparable regulons in eukaryotes, in which related but different elements are present in mRNA targets scattered among the chromosomes. The same strategy exploited by the virus can be pitted against the virus, as targeting the element can simultaneously inhibit the expression of all sgRNAs. In-depth elucidation of virus translation control elements, their binding proteins, and their mechanisms can lead to novel therapeutic approaches to reduce virus replication and pathogenicity.

## Data Availability

There are no original data in the paper.
